# Clinical efficacy and safety of traditional Chinese patent medicine for hyperthyroid heart disease: study protocol for a systematic review and meta-analysis

**DOI:** 10.1097/MD.0000000000013076

**Published:** 2018-11-09

**Authors:** Qing Wang, Chun Li, Sha Di, Lin Han, Linhua Zhao, Xiaolin Tong

**Affiliations:** a Guang’anmen Hospital of China Academy of Chinese Medical Sciences; b Beijing University of Chinese Medicine; c Dongzhimen Hospital of Beijing University of Chinese Medicine, Beijing, China.

**Keywords:** hyperthyroid heart disease, protocol, systematic review, traditional Chinese patent medicine

## Abstract

**Background:**

Hyperthyroid heart disease (HHD), one of the most common complications of hyperthyroidism, is a serious public health problem due to the direct toxic or indirect effects of excessive thyroid hormone on the heart, resulting in high mortality and increasing health care costs. Traditional Chinese patent medicines (TCPMs), developed by combining modernized pharmaceutical technologies with ancient TCM theories, have been widely used in the treatment of HHD. However, the safety and efficacy of TCPMs used in patients with HHD has been uncertain and there has been no standard clinical trial published to confirm this. Thus, we conduct a study to evaluate the safety and efficacy of TCPMs for HHD.

**Methods:**

The reference lists of randomized controlled trials and 8 electronic databases will be independently and systematically searched by 2 review authors in August 2018. Four English databases [EMBASE, PubMed, National Guideline Clearinghouse (NGC), and Cochrane Central Register of Controlled Trials (CENTRAL)] and 4 Chinese databases [Chinese Biomedical Literature Database (CBM), Chinese National Knowledge Infrastructure [CNKI], Wanfang Database, and VIP Database] will be included. The primary outcomes will be assessed according to the effective rate of treatment, electrocardiogram, and thyroid hormone levels. Data synthesis will be precisely computed using the RevManV5.3 software when a data-analysis is allowed. Methodological quality will be assessed according to Cochrane Handbook.

**Results:**

This study will provide a high-quality synthesis of current evidence of TCPMs for HHD from different aspects, including the clinical symptoms, thyroid hormone levels, and ECG changes.

**Conclusion:**

The conclusion of this systematic review will provide evidence to prove whether TCPMs are effective therapeutic intervention for patient with HHD.

## Abstract 2

Supplemental Digital Content is available in the text

## 1 Introduction

Hyperthyroidism is a group of clinical syndromes in which thyroid gland hyperfunction leads to excessive synthesis and secretion of thyroid hormones, resulting in increased excitability and hypermetabolism in the nervous, circulatory, and digestive systems. The incidence rate includes 2% to 5% of the population, second only to diabetes and osteoporosis. Notably, hyperthyroidism is a clinically common endocrine disease.^[1]^ Hyperthyroid heart disease (HHD) is one of the most common complications of hyperthyroidism. It occurs as a result of direct toxicity or indirect effects of excessive thyroid hormone on the heart, causing a series of cardiovascular system signs and symptoms, such as tachycardia, elevated cardiac output, atrial fibrillation (AF), heart failure, and metabolic changes. The incidence of HHD includes 10% to 22% of patients with hyperthyroidism, most notably 28.6% of those over 60 years old.^[2]^ It increases mortality in patients with hyperthyroidism by 20%, particularly among elderly patients.^[3]^ Among the signs and symptoms of HHD, AF is the primary manifestation and has high risks of thromboembolism and heart failure, which greatly affect patients’ quality of life and safety.^[4]^ Importantly, it is the leading cause of death in hyperthyroidism.^[5]^

Thus far, there has been no unified diagnostic standard for HHD.^[6]^ Clinically, HHD is often initially diagnosed as hyperthyroidism, accompanied by one or more cardiac abnormalities (e.g., sinus tachycardia, atrial fibrillation, cardiac enlargement, and/or heart failure). The goal is to exclude heart disease caused by other etiologies; if cardiovascular symptoms and signs largely disappear or are significantly reduced after the cure of hyperthyroidism, the disease can then be diagnosed. Because of long-term high cardiac output, high heart rate, high pulse pressure difference, and high oxygen volume, untreated or long-term HHD can result in a variety of complications, including the following left ventricular enlargement, reduced ventricular diastolic function, reduced arterial compliance, and left atrial enlargement, as well as AF and pulmonary hypertension; these eventually lead to stroke, angina pectoris, myocardial infarction, heart failure, and sudden death. Notably, HHD and its complications seriously threaten patients’ health,^[7]^ which emphasizes the importance of early diagnosis and early treatment of HHD patients. In treatment of HHD, the primary disease is treated first, in order to control hyperthyroidism. There are 3 main methods: antithyroid drugs, radioactive ^131^ I, and surgical treatment. Other methods, such as interventional embolization therapy and Chinese medicine, have also been used in recent years. On this basis, comprehensive treatment measures are taken to jointly reduce ventricular rate, correct heart failure, and correct electrolyte imbalance. Although there is evidence that anticardiovascular disease drugs can alleviate heart symptoms or ECG results, adverse events may limit the use of these drugs. For example, when patients take the arrhythmia drugs, the side effects are obvious, including arrhythmia,^[8]^ and other serious complications.^[9]^

One of the most appreciable distinctions between China and the West in treating HHD is the use of Traditional Chinese patent medicine (TCPM) as an adjunct therapy. In recent years, TCPMs have become increasingly popular in China. Proprietary TCPMs are developed by combining modernized pharmaceutical technologies with ancient TCM theories. Refined dosage forms and relative standardization in composing the main effective components are considered advantages of TCPMs compared with herbal decoctions.^[10]^ Currently, many TCPMs are used for the prevention and treatment of HHD; these TCPMs have been used in clinical practice for many years in China. Although the included TCPMs vary in their herbal components, they form part of a “group” of herbal medicines with anti-hypermetabolism in circulatory system effects designed to prevent HHD and decrease the hypermetabolism in circulatory system. The main therapeutic principle in the field of TCM includes fortifying qi, clearing heart heat, calming the mind, nourishing yin, activating blood, and dissolving stasis.

Many patients with HHD are willing to choose TCPMs, because their disease has not yet reached the stage that requires long-term use of anticardiovascular disease drugs, or because the application of anticardiovascular disease drugs cannot fully relieve their symptoms. In addition, the use of anticardiovascular disease drugs is often accompanied by adverse events, whereas TCPMs are conveniently administered and easier to tolerate. Pharmacological investigations have indicated that TCPMs have beneficial effects on the relief of arrhythmia, palpitations, chest tightness, heartache, shortness of breath, fatigue, dizziness, and insomnia.^[11]^ Although several studies have suggested that TCPMs or TCPMs combined with antithyroid drugs are effective for the treatment of HHD, few systematic reviews have been published regarding the effects of TCPMs for treating HHD. We performed a systematic review and meta-analysis to assess the strength of the current evidence to support the efficacy and safety of TCPMs for the treatment of HHD, which might constitute a complementary therapy for HHD.

## 2 Methods

### 2.1 Inclusion criteria for study selection

#### 2.1.1 Types of studies

All the randomized controlled trials (RCTs) that investigated the effect of TCPM for the treatment of HHD will be included. Nonrandomized clinical studies and case studies will be excluded. No publication type of restriction or writing language will be applied in this study.

#### 2.1.2 Types of patients

Trials involving participants with HHD will be included without limitations of age, sex, education status, or ethnic background. Patients with HHD should be diagnosed by physicians based on the hyperthyroidism diagnostic standard established by the American Thyroid Association (ATA), accompanied by one or more cardiac abnormalities.

#### 2.1.3 Types of interventions

According to the treatment guidelines of hyperthyroidism, the participants in the experimental group will be treated with the therapeutic intervention of conventional western medical treatment (no restrictions on antithyroid drugs, radioactive ^131^ I, or surgical treatment) and TCPM, whereas the controlled group will be treated with the conventional western medical treatment (no restrictions on antithyroid drugs, radioactive ^131^ I, or surgical treatment). Or the participants in the experimental group will be treated with the therapeutic intervention of conventional western medical treatment (no restrictions on antithyroid drugs, radioactive ^131^ I or surgical treatment), anticardiovascular diseases drug, and TCPM, whereas the controlled group will be treated with the conventional western medical treatment (no restrictions on anti-thyroid drugs, radioactive ^131^ I, or surgical treatment) and anticardiovascular diseases drug. Or the participants in the experimental group will be treated with the therapeutic intervention of conventional western medical treatment (no restrictions on anti-thyroid drugs, radioactive 131 I, or surgical treatment) and TCPM, whereas the controlled group will be treated with the conventional western medical treatment (No restrictions on antithyroid drugs, radioactive ^131^ I, or surgical treatment) and anticardiovascular diseases drug. The course of treatment will be greater than or equal to 2 weeks.

#### 2.1.4 Types of outcome measures

##### 2.1.4.1 Primary outcomes

Primary outcomes are as follows:

(1) effective rate of treatment
(2) electrocardiogram (ECG)
(3) thyroid hormone levels (TSH, FT3, FT4, TT3, TT4)

##### 2.1.4.2 Secondary outcomes

Secondary outcomes are as follows:

(1) clinical symptoms
(2) adverse events

The included studies must include one or more of the primary outcomes.

### 2.2 Search methods for the identification of studies

#### 2.2.1 Electronic searches

Two research members (QW and CL) will electronically and independently search 4 English databases [EMBASE, PubMed, National Guideline Clearinghouse (NGC), and Cochrane Central Register of Controlled Trials (CENTRAL]] and 4 Chinese databases [Chinese Biomedical Literature Database (CBM), Chinese National Knowledge Infrastructure (CNKI), Wanfang Database, and VIP Database) from their inception to August 2018. The searched items will be used as follows: HHD, TCPM, and RCTs. The same terms will be searched in the Chinese databases. The data will be retrieved with the combination of medical keywords and uncontrolled terms. The detailed retrieval strategy of PubMed database will be shown in the supplemental digital content (Appendix A), and will be constantly modified by searching other databases.

#### 2.2.2 Searching other resources

Meta-analysis of RCTs and recently relevant systematic reviews will be electronically searched. Furthermore, the related conference proceedings and reference list of eligible studies will be particularly searched to avoid the eligible trials as well.

### 2.3 Data collection and analysis

#### 2.3.1 Selection of studies

**Figure 1:**
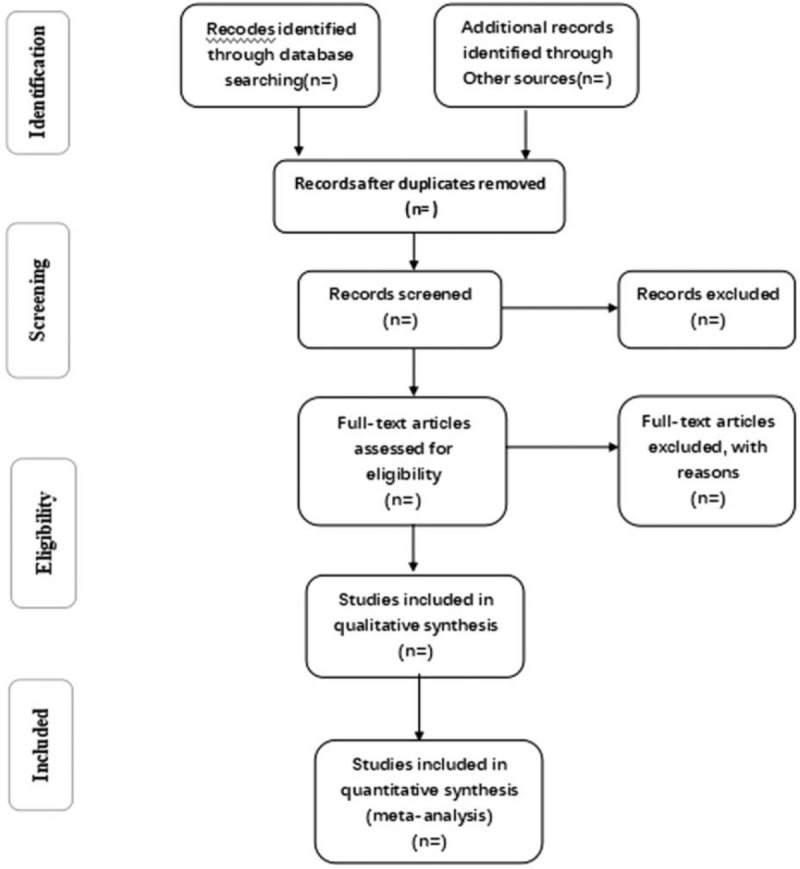
Flow diagram of study selection process.

Reviewers will receive professional training to be familiar with the background, objective, and process of this review. Relevant studies obtained from the databases will be uploaded to a literature management system of EndnoteX8. Two search reviewers will independently carry out the selection and record their decisions with a standard eligibility form by screening the titles and abstracts of the retrieved articles. They will read the whole article later to meet the requirements and check the final inclusion of references. Any disagreement between 2 reviewers about the inclusion of studies will be resolved through discussion. If the discussion cannot reach a consensus, the third search reviewer will make a final decision of the selection. Details of the selection process of studies will be shown in a PRISMA flow chart (Fig. 1).

#### 2.3.2 Data collection and management

Two search reviewers will read all the included articles and independently collect data via a standardized eligibility form. General information of the retrieved articles will be extracted, including the first author, year of publication, study design, sample size, age, sex, duration and severity of disease, intervention, and treatment applied in the control group. Outcome measures and further information such as results of the study, adverse events, and conflicts of interest will be extracted as well. Any divergence of the data extraction will be discussed and resolved between 2 search reviewers. The final results of the data extraction will be collated by the arbiter. When the data are insufficient or ambiguous, one of the reviewers will contact the original author to acquire additional and detailed information by telephone or e-mail.

#### 2.3.3 Assessment of risk of bias in included studies

Two review authors will independently assess the risk of bias of the included studies according to the Cochrane Collaboration's tool provided by Cochrane Handbook V.5.1.0, which involves the following 7 domains: blinding of participants, randomize sequence generation, allocation concealment, personnel and outcome assessors, selective reporting, incomplete outcome data addressed, and other issues. The result of the evaluated domains will be classified into 3 levels: low risk, high risk, and unclear risk. Any discrepancies will be resolved through team discussion to reach an agreement. If necessary, a third reviewer will be consulted to confirm the details.

#### 2.3.4 Measures of treatment effect

For dichotomous outcomes, a relative risk (RR) with 95% confidence interval (95% CI) will be used to identify the effect of treatment. For continuous and integrated data, a mean difference (MD) will be presented with 95% CI to evaluate the extracted data.

#### 2.3.5 Unit of analysis issue

Only the data obtained from the meta-analysis and RCTs studies will be adopted. If the data of crossover trial are involved, we will merely use the first-phase data. If the unit of analysis has multiple time points to observe, we will divide the time point observation into 2 terms: a short term (within 1 month) and a long term (over 1 month).

#### 2.3.6 Dealing with missing data

If it is possible, we will try to come in contact with the first author via e-mail or telephone to request for the inadequate and missing data. If it cannot work, the analysis will be built only with the available data and potential effect of the missing data.

#### 2.3.7 Assessment of heterogeneity

According to the guideline of Cochrane Handbook V.5.1.0, the heterogeneity of the results can be evaluated with the x^2^ test (a = 0.1). If the value determined by *I*^2^ exceeds 50%, the heterogeneity among trials will be considered to be significant. Subgroup analysis will be carried out to explore the correlative causes of heterogeneity.

#### 2.3.8 Assessment of reporting biases

If sufficient trials are included in the study (>10 trials), visual asymmetry on a funnel plot will be utilized to detect reporting bias and the funnel plot asymmetry will be evaluated with the use of the test of Egger regression.

#### 2.3.9 Data synthesis

RevMan software V5.3 from Cochrane Collaboration will be used to compute the data synthesis and conduct meta-analysis when suitable. The analysis with a fixed-effect model will be performed to calculate the RR and MD with low heterogeneity (*I*^2^ < 50%). If not, a random-effect model will be employed to synthesize the data.

#### 2.3.10 Subgroup analysis

Owing to the inconsistency among participant characteristic, detailed interventions, and outcome measures, subgroup analysis will be performed if the amount of included trials is sufficient (at least 10 trials). Subgroup analysis is carried out to explore the potential causes of the heterogeneity.

#### 2.3.11 Sensitivity analysis

We will perform sensitivity analysis to determine the quality and robustness of results according to the following criteria: sample size; analysis issue (such as the impact of missing data); methodological quality.

#### 2.3.12 Dissemination and ethics

The results of this systemic review will indicate the efficacy and safety of TCPM for HHD. This review will be disseminated through publication in a peer-reviewed journal and presentation at a relevant conference. It is not necessary for a formal ethical approval because the data are not individualized.

#### 2.3.13 Grading the quality of evidence (Summary of evidence)

The Grading of Recommendations Assessment, Development, and Evaluation (GRADE) is a tool to evaluate the quality of primary outcome. The level of evaluation will be divided into 4 types: high, moderate, low, or very low.

If quantitative synthesis is not appropriate, we will conduct a qualitative study of systematic review.

## 3 Discussion

HHD is a major health issue, the incidence of which occurs worldwide. Although medical technology has improved rapidly in the treatment of HHD, the mortality and morbidity of HHD continue to increase. During the development of HHD, thyroid hormone can directly act on vascular smooth muscle cells, producing endothelial nitric oxide to dilate peripheral blood vessels and reduce peripheral resistance; moreover, it reduces renal perfusion pressure and activates the renin angiotensin system (RAS), causing water-sodium retention in the body. Thyroid hormone can promote the production of erythropoietin, increase the red blood cell content in blood, raise the blood volume of the heart, and cause high cardiac output. Long-term high thyroid hormone levels in the human body can cause left ventricular hypertrophy and pulmonary hypertension, as well as reductions in arterial compliance and diastolic function in the heart, eventually leading to the onset of clinical HHD.^[12]^ Thyroid hormone can also damage the body's coagulation mechanism, resulting in hypercoagulation and low fibrinolysis.^[13]^ In the past few decades, although many anticardiovascular disease drugs have emerged, Western medicine has become the world's leading medical treatment. It can partially improve the patient's heart function and alleviate clinical symptoms, but negative effects are obvious, and the overall effect is unsatisfactory.

To resolve these problems, TCPMs, a set of modern prescriptions derived from TCM, have been widely applied for the treatment of HHD in China, with better therapeutic efficacy and fewer side effects. Several published studies have demonstrated that TCPMs could have an effective impact on the treatment of HHD with respect to alleviation of symptoms; these can be used to treat ventricular premature beats, arrhythmia, and premature ventricular contractions, as well as effectively improve microcirculation and improve myocardial contractility.^[14]^ Pharmacological studies have shown that TCPMs can protect against myocardial mitochondrial peroxidative damage, reduce levels of inflammatory factors (e.g., TNF-a) in myocardial tissue, and inhibit cardiomyocyte apoptosis. Importantly, TCPMs have clear cardiovascular protective effects and exhibit good safety.^[15]^ Because TCPMs are increasingly used in the treatment of clinical endocrine diseases in China, an increasing number of studies have been performed regarding their effectiveness and mechanisms in recent years.^[16,17]^ TCPMs have been repeatedly tested in clinical trials and confirmed to be effective and safe, before release to the market. However, relevant evidence from clinical studies and the exact mechanisms of TCPMs in the treatment of HHD require further exploration. In addition, in the English literature, there is no meta-analysis or systematic review regarding the efficacy and safety of TCPMs in the treatment of HHD. Therefore, the objective of this systematic study was to evaluate the efficacy and safety of TCPMs in the treatment of HHD.

## Author contributions

**Conceptualization:** Qing Wang, Linhua Zhao, Xiaolin Tong.

**Data curation:** Chun Li.

**Formal analysis:** Sha Di.

**Funding acquisition:** Xiaolin Tong.

**Guarantor of the review:** Linhua Zhao, Xiaolin Tong.

**Investigation:** Chun Li.

**Methodology:** Qing Wang, Sha Di, Lin Han.

**Project administration:** Linhua Zhao, Xiaolin Tong.

**Writing – original draft:** Qing Wang.

**Writing – review & editing:** Qing Wang.

## Supplementary Material

Supplemental Digital Content
