# Supplementary material for: Clinical efficacy and safety of traditional Chinese patent medicine for hyperthyroid heart disease: study protocol for a systematic review and meta-analysis
Source: Medicine (Baltimore). 2018 Nov 9;97(45):e13076. doi: 10.1097/MD.0000000000013076 (PMC6250447; doi:10.1097/MD.0000000000013076)
Supplement: Supplemental Digital Content [file medi-97-e13076-s001.doc]

Appendix A. The detailed retrieval strategy of PubMed database.

(graves [Title/Abstract] OR hyperthyroidism [Title/Abstract] OR hyperthyreosis [Title/Abstract] OR goiter [Title/Abstract] OR thyrotoxicosis [Title/Abstract]) AND (heart [Title/Abstract] OR cardio [Title/Abstract]) AND (TCM [Title/Abstract] OR TCPM [Title/Abstract] OR Traditional Chinese medicine [Title/Abstract] OR Tang [Title/Abstract] OR San [Title/Abstract] OR Chinese patent drug [Title/Abstract] OR Wan [Title/Abstract] OR Capsule [Title/Abstract] OR Chinese patent medicine [Title/Abstract] )
